# Cross-Talk between Cadmium and Selenium at Elevated Cadmium Stress Determines the Fate of Selenium Uptake in Rice

**DOI:** 10.3390/biom9060247

**Published:** 2019-06-24

**Authors:** Muhammad Umer Farooq, Zhichen Tang, Tengda Zheng, Muhammad Ahsan Asghar, Rui Zeng, Yang Su, Hla Hla Ei, Yuanke Liang, Yujie Zhang, Xiaoying Ye, Xiaomei Jia, Jianqing Zhu

**Affiliations:** 1 Demonstration Base for International Science & Technology Cooperation of Sichuan Province, Rice Research Institute, Sichuan Agricultural University, Chengdu 611130, Sichuan, China; umerpbguaf@gmail.com (M.U.F.); tangzhichen0516@gmail.com (Z.T.); 13089068357@163.com (T.Z.); zengrui829@163.com (R.Z.); imsuxiaomeng@163.com (Y.S.); hlahlaeidoaa@gmail.com (H.H.E.); ykliang77@163.com (Y.L.); rachel.zhangyujie@outlook.com (Y.Z.); yeyixuana@163.com (X.Y.); jessicamei2372@sina.com (X.J.); 2 College of Agronomy, Sichuan Agricultural University, Chengdu 611130, Sichuan, China; ahsanasghar2017@outlook.com; 3 Dujiangyan Agricultural and Rural Bureau, Dujiangyan 611830, Sichuan, China

**Keywords:** cadmium and selenium crosstalk, accumulation trend, selenium hyper-accumulation, selenium-rich rice, non-selenium rich rice

## Abstract

Cadmium (Cd) is a well-known metal imposing threats to human health, and it can be accumulated in polished rice over the permitted range of 0.2 mg kg^−1^ (GB 2762-2017). It has been reported that selenium (Se) application decreases Cd uptake. Se-rich diets have gained attention recently, but the potential of Se-rich rice in mitigating Cd stress needs further investigation. In this study, a pot experiment in the field was conducted to assess the influence of environmental factors and exogenous split application of Se on the nutritional status of rice under Cd stress. The results indicated that the increased fertilizer treatment in soil bulk linearly increased the metal content in rice grains. Approximately 50–70% of metal was recovered in rice tissues, while 5–20% of the metal that was applied leached down into the soil. A Se concentration of 0.4 mg kg^−1^ could significantly improve the total Se content in grain and mitigate Cd toxicity (1 mg kg^−1^) below the permitted range. Panicles and roots were more active for total Se accumulation in Se-rich and non-Se-rich rice, respectively. Polishing and milling operations can significantly reduce the Cd content, as rice bran in rice tissues accumulated most of the metal’s residues. The late matured rice cultivars consumed more heat units, and more metal contents were found in them. Collectively, it was found that Se can mitigate Cd toxicity, but the rice cultivation at T_2_ (high Cd; 2 mg kg^−1^ and Se; 1 mg kg^−1^) increased the metal uptake capability and health-risk index in polished rice, with its Se content heightened over permitted range of 0.04 to 0.30 mg kg^−1^ (GB/T 22499-2008). However, further molecular studies are required, in order to completely access the inverted Se accumulation behavior in rice tissues at high Cd soil stress.

## 1. Introduction

Cadmium (Cd) is regarded as a Group I carcinogen for humans [1]; it is the third most toxic metal after lead and mercury [2], and is widespread in the atmosphere and soil [3]. In China, 13,000 hectares of farmland is contaminated by Cd [4] due to various sources (public Cd disposal waste, phosphate application, sewage sludge, and industrial emissions) [3,5]. The half-life of Cd is 30 years, which leads to 50–70% accumulation of element in human body with age, especially in kidneys [6] and lungs [7]. Rice (*Oryza sativa* L.) is one of the world’s leading grains and a major source of food for more than half of the world’s population [8]; it contributes 55–80% toward a person’s total calorie intake [9]. Rice can accumulate high levels of Cd [10]. In the 1950s, the people of Japan were afflicted by a disease called “Itai-Itai” with symbols of calcium loss in bones, anemia, and severe muscle pain due to the utilization of rice grown in Cd-contaminated industrial water. In the past, due to a lack of food and technical constraints, scientists mainly focused on high yield and disease resistance varietal development. With the advent of time, rice quality has gradually become the prime focus of the research community. Therefore, effective strategies need to be adopted to avoid health risks. 

Selenium (Se) has many effects on the growth and development of plants. It regulates various physiological processes and attenuates the toxic effects of heavy metals and free radicals [11,12,13]. Soil is the main source of nutrition in plants, and effectiveness of soil Se absorption in rice depends on many factors, such as soil Se contents, Se forms, the alkalinity/acidity of soil, and metal–ion interactions [14,15,16]. Selenium is considered as a double-edged sword with narrow safe boundaries. Its deficiency and surplus quantity in the body can affect human health [17]. The recommended daily Se intake for adults varies (40 to 300 μg/day) between agencies [18,19]. The safe range of this element needs to be controlled in polished rice [20]. Selenium has antagonistic effects on Cd mitigation, as disclosed by many hydroponic experiments [12,21,22]. However, studies describing the potential of Se-rich rice in mitigating Cd stress are rather limited. Rice biofortified with beneficial trace elements could rather be assessed for the reclamation of Cd-affected areas. The performances regarding the known behavior of these elements could be entirely different in actual field conditions due to multiple environmental and epigenetic effects.

A promising area for an initial study always exists, and there is a dire need to extensively explore the interactive effects of Cd and Se on rice nutritional status. For this purpose, natural Se-rich and non-Se rich rice cultivars were chosen. Our specific aims were (I): to accurately assess the metal’s accretion behavior in different components (soil, roots, stem, leaves, panicle, husk, panicle straw, rice bran, embryo, and endosperm), (II) to determine the health risks associated with exogenous fertilizer, and (III) to elucidate the manipulative role of heat units on the nutritional status of rice.

## 2. Materials and Methods

A soil culture potted experiment was conducted in the research area of Rice Research Institute, Sichuan Agricultural University (Chengdu, China). Paddy soil was collected from five different locations in the same field and homogenized to make a blend to access the basic physiochemical properties of soil (Table 1). 

### 2.1. Experimental Material

Three rice cultivars—5097A/R2035 (natural High Se-rich), 2057A/R881(natural moderate Se-rich), and GangYou 725 (non-Se-rich)—were provided by the Demonstration Base for the International Science and Technology Cooperation, Rice Research Institute of Sichuan Agricultural University (Chengdu, China). GangYou725 was used as positive control, while the performance of moderate (2057A/R881) and high (5097A/R2035) Se-rich rice cultivars were compared to it. Using heterosis [23], the material was cross-bred over years of generations in order to have a bioaccumulation effect on Se. The breeding process of these lines can be seen in our published reports [24,25]. The Se-rich rice groups 2057A/R881 and 5097A were tested by the Rice Testing Centre of the Ministry of Agriculture, and the Se content in polished rice was found to have accumulated 0.069 mg kg^−1^ and 0.14 mg kg^−1^ (GB/T 5009.93-2010), respectively, which meets the national standard for a Se-rich paddy: that is, 0.04–0.30 mg kg^−1^ (GB/T 22499-2008). The non-Se-rich rice (GangYou 725) was tested and found to accumulate 0.007 mg kg^−1^ Se in polished rice. The Se content of non-Se-rich rice was below the standards for a Se-rich paddy, and hence was defined as non-Se-rich rice.

### 2.2. Planting Conditions

A nursery of the test material was grown in normal soil. All the required conditions (irrigation, hoeing, fertilization, weeding) needed for the normal growth of rice were maintained until the nursery entered in a two-leaf stage. Pots were cleaned and equally filled with 10 kg of nutrient-deficient soil (which was obtained by digging soil 3 ft below the surface) and placed in the field to completely assess the interactive role of environmental factors on the nutritional status of rice. Plantlets with uniform health were selected from the nursery and transplanted at a rate of three seedlings per pot. The water table was maintained 5 cm in pots throughout the growth period. The material was randomly assigned in pots using randomized complete block design (RCBD) to avoid the horizontal and vertical soil fertility effects on the nutrient’s uptake. The seedling was allowed to establish root systems in pots, and after seven days of transplantation, nutrient treatments were applied. 

### 2.3. Nutrient Treatments Used in the Experiment

Cadmium and Se were purchased in chemical compound form as sodium selenite (Na_2_SeO_3_) and cadmium chloride (CdCl_2_.2½H_2_O). The Cd and Se content in the compounds were determined using formula:

(1) $$Element\;in\;compound=\frac{Atomic\;mass\;of\;Element}{molecular\;weight\;of\;compound\;}\times 100$$

### 2.4. Selenium and Cadmium Stress Treatment Groups

To observe the bioaccumulation effect of trace elements in different cultivars, two levels of Se and Cd nutrients i.e., T_1_ (Se; 0.4 and 1 mg kg^−1^ soil), and T_2_ (Cd; 1 and 2 mg kg^−1^ soil) were applied to access differences between non-Se-rich (Gangyou725) and Se-rich rice groups (5097A/R2035, 2035/R881) (Table 2).

### 2.5. Sample Preparation and Metal Analysis

The material pre-treatment of stem, leaves, and panicle parts (panicle straw, husk, rice bran, embryo, endosperm) was made by the previously described method [24]. Roots were surface washed twice with double deionized water to separate the impurities attached on the root surface and dried at 85 °C in an air dryer for three consecutive days to achieve constant weight. Then, the dried roots were ground with a mortar and pestle to get the residue for analysis. The plant samples (roots, stem, leaves, and panicle parts) were dried, weighed (0.1 g), placed in a glass vial, and then digested using 15 mL of diacid HNO_3_:HClO_4_ acid solution (9:1, *v/v*) at a temperature of 190 °C on an electric hot plate (EH20A Plus, Labtech, Hopkinton, MA 01748, USA) until the solution turned whitish. Then, the solution was evaporated and reduced to 1 mL. The Se at this level needs to be reduced from Se^6+^ to Se^4+^ to get the total Se (all Se^4+^) content in digested samples. Hence, the digestion method for Se was further different.

#### 2.5.1. Determination of Total Se in Samples

Then, the digested samples were diluted by 5 mL of HCl: H_2_O solution (1:1, *v/v*) to reduce Se^6+^ into Se^4+^ for total Se determination. The solution was digested again at 160 °C until the solution again turned whitish with a volume less than 1 mL. Then, the solution was diluted three times with 5% HCl solution and filtered by a 0.02-µm membrane filter paper to retain a final volume of 10 mL in 15-mL centrifugal tubes. Then, the final filtered solution was analyzed by RGF-6800 (Bo Hui Co., Ltd., Beijing, P. R. China). The results were expressed as mg kg^−1^. 

#### 2.5.2. RGF-6800 Parameter Settings

We set the following parameters: negative high voltage: 340 V; lamp current: 100 mA; atomization temperature: 80 °C; high furnace: 8 mm; carrier gas flow rate: 500 mL/min; shielding gas flow rate: 1000 mL/min; measurement methods: standard curve; reading: peak area; delay time: 1 s; reading time: 15 s; charging time: 8 s; sample size: 2 mL. Se content (mg kg^−1^) was calculated using the formula:

(2) $$Se\;content=\frac{\left(C-C_{0}\right)\times V\times 1000}{m\times 1000\times 1000}$$

where *C* is the sample measured concentration of the digestive solution (ng mL^−1^); *C_0_* is a concentration of the blank control group (ng mL^−1^); *m* is the mass of samples; and *V* is the total volume of digestive solution. 

#### 2.5.3. Determination of Cd Contents in Samples

Then, the evaporated solution left with 1-mL volume (Section 2.5) was diluted three times with 5% HCl solution and filtered by a 0.02-µm membrane filter paper to retain a final volume of 10 mL in 15-mL centrifugal tubes. Then, the final filtered solution was analyzed using iCE^TM^-3300 (Thermo, 168 Third Avenue Waltham, MA USA). The results were expressed as mg kg^−1^.

#### 2.5.4. iCE^TM^-3300 Machine Parameters and Lamp Settings

The Thermo Solaar software by iCE^TM^-3300 (Thermo, 168 Third Avenue Waltham, MA, USA) was used to analyze the Cd content in the sample and parameter setting was as follows: negative high voltage: 260 V; lamp current: 60 mA; ash temperature: 300 °C; atomization temperature: 900 °C; high furnace: 8 mm; carrier gas flow rate: 400 mL/min; shielding gas flow rate: 900 mL/min; measurement methods: standard curve; reading: peak area; delay time: 1 s; reading time: 15 s; charging time: 8 s; sample size: 1 mL. Cd content (mg kg^−1^) was calculated using the formula:

(3) $$Cd\;content=\frac{\left(C-C_{0}\right)\times D\times V\times 1000}{m\times 1000\times 1000}$$

where *C* is the sample measured concentration of the digestive solution (ng mL^−1^); *C_0_* is a concentration of the blank control group (ng mL^−1^); *D* is the dilution factor; *m* is the mass of samples; and *V* is the total volume of digestive solution. 

### 2.6. Preparation of Soil Powder

Soil samples were collected from three spots in one pot using a T-shaped steel hollow rod. The instrument collected a soil sample from two depths. The upper layer of soil collected from the instrument was defined as 10 cm, and a lower layer was defined as 20 cm. Then, both layers of soil were separately collected in polyethene plastic bags and air dried under natural conditions. After two weeks of drying at room temperature, the dried soil samples were then broken down into small pieces using a hammer and ground with a pestle and mortar into powder. Last, the samples were sieved over a 100-grade sifter to get a fine powder and stored in polythene bags for further analysis.

#### Determination of Se and Cd in Soil Powder

A 0.1-g soil sample was placed in 25-mL PolyTetraFluoroEthylene (PTFE) digestive tubes (Aurum Resources Inc., Maple Ridge, BC V2W 1C2, Canada). Then, 2–3 drops of water were added to make a paste of viscous clot that could easily homogenize the sample with the acid. Then, 10 mL of diacid HClO_4_: HNO_3_ solution (1:10, *v/v*) along with 5 mL of HF (hydrofluoric acid) was added into it. HF helps clean the soil impurities and make digestion feasible. The samples were digested for 8 h at a temperature of 160 °C on an electric hot plate (EH20A Plus, Labtech, Hopkinton, MA 01748, USA) until the solution turned clear, light yellow, and reduced to 1 mL. 

Then, the tubes that were not digested completely were separated out and allowed to cool at room temperature. Then, 3 mL of diacid HClO_4_: HNO_3_ solution (1:10, *v/v*) along with 1 mL of HF solution was added and tubes were again digested at a temperature of 200 °C for the next 8 h until the solution turned clear, light yellow, and reduced to 1 mL. Then, the digested samples were allowed to cool at room temperature and diluted with double-deionized water to retain a final volume of 50 mL in a conical flask, following filtration with 0.02-µm membrane filter paper. Then, Se contents were analyzed using dual channel atomic fluorescence analyzer (RGF-6800, Bo Hui Co., Ltd., Beijing, P. R. China) using standards (GB-5009.93-2017). Meanwhile, atomic absorption spectrophotometry by iCE^TM^-3300 (Thermo, 168 Third Avenue Waltham, MA, USA) was used for the determination of Cd (GB-5009.15-2014). Soil pH was measured using a pH meter (Accumet^®^ AP115, 168 Third Avenue Waltham, MA, USA). 

### 2.7. Estimation of Heat Units for Each Respective Stage

The rice developmental stages were recorded at an appropriate time in order to assess the influence of environmental factors (especially heat units) on the nutritional status of rice. The dates were recorded for morphologically distinct stages (tillering, 50% heading, flowering, 50% maturity, complete maturity, and harvesting), and the heat units consumed by rice cultivars to reach a respective stage were estimated using formula [26] with little modifications. 

(4) $$Heat\;Units=\frac{Max\;Temp\;-\;Min\;Temp\;of\;a\;day}{2}-Base\;Temp\;of\;Crop$$

The temperature was taken in degrees Celsius (°C) while the base temperature of the crop was set as 10.

### 2.8. Statistical Analysis

For elemental detection samples were collected from two experimental replicates and two biological repeats (all rice tissues (soil, roots, stem, leaves and panicle parts) were analyzed. However, the iCE^TM^-3300 (Thermo, 168 Third Avenue Waltham, MA, USA) and RGF-6800 (Bo Hui Co., Ltd., Beijing, P. R. China) machine further analyzed each sample twice (two technical repeats). In the end, the data for Se and Cd contents were extracted using specified formulas for Cd and Se content estimation in the material and methods section (Section 2.5.2 and Section 2.5.4), averaged, and mentioned in tables as mgkg^−1^ (Supplementary Tables S3–S5). The overall replicates include vast sample size (three varieties, two experimental replicates (all rice tissues), two biological repeats, and two technical replicates by machine for each tested sample). However, the samples were arranged randomly in the field conditions to avoid the epigenetic environmental effects, as well as vertical and horizontal soil fertility effects on the nutritional aspects of rice. Statistix 8.1 (User’s Manual. (2003) Analytical Software, Tallahassee) was used to estimate the variance differences (ANOVA). We observed multiple factors in the experimental design (nutrients (Cd, Se), including different varieties (V1, V2, V3), different stress levels (T0, T1, T2), and their effects on rice tissues. Hence, two-factor factorial analysis design (varieties × stress, treatment × stress and interaction effects with all pairwise possible combinations) was implemented on rice tissues (Table 3). The least significance difference (LSD) test was performed to compare means at the 5% probability level. Then, the data were fitted into linear y = ax + b models to analyze the correlations using Microsoft Office Excel 2016. Moreover, Origin 8.0 (OriginLab Corporation, Northampton, MA, USA) was used for the graphical presentation of data.

## 3. Results and Discussion

### 3.1. Cd and Se Activity in the Soil

**Figure 1:**
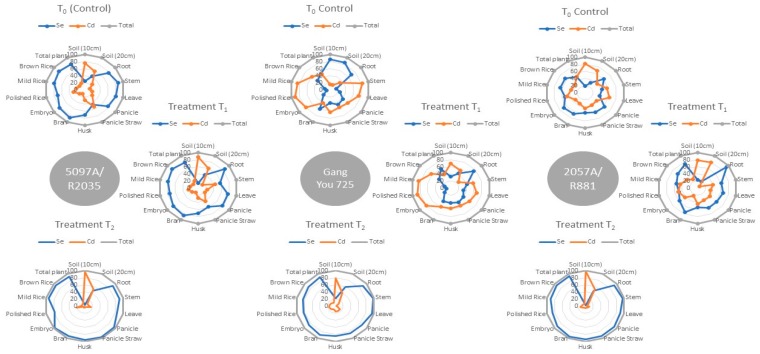
Web diagram indicating the differences between Cd and Se uptake in percentage (three varieties, three treatment levels, different plant parts). Note: T_0_ Control (natural soil conditions), T_1_ (Se; 0.4 mgkg^−1^, Cd; 1 mgkg^−1^), T_2_ (Se; 1 mgkg^−1^, Cd; 2 mgkg^−1^). 5097A/R2035: natural high Se-rich rice, GangYou 725; natural non-Se rich rice, 2057A/R881: natural moderate Se-rich rice.

Soil is the medium withholding capacity of nutrients that are necessary for plant’s life. Different layers of soil behave differently to the nutrient’s mobilization. The results of present study indicated that the translocation of Cd was limited with approximately 75% to 82% Cd content in upper layer (10 cm), while the lower layer of soil (20 cm) was found to have more Se content. Se-rich rice cultivars were more sensitive to Se uptake, as Se and Cd content of approximately 25% to 33% and 66% to 76% were observed in their pot’s soil, respectively. Out of the total Se (25% to 33%) found in soil, approximately 30% to 42% and 17% to 24 % of Se was found in the lower (20 cm) and upper layer (10 cm) of soil, respectively (Figure 1, 5097A/R2035, 2057A/R881: T_0_). Inversely, the Se and Cd contents were 85.12% and 14.87% in non-Se rich rice (GangYou 725) pot’s soil (Figure 1, GangYou 725~T_0_). The exogenous Se application (1 mg kg^−1^) significantly reduced the Cd uptake and sequestration in rice tissues (Figure 1, 5097A/R2035, GangYou 725, and 2057A/R881~T_2_). The specific architecture of breeding material unveiled that under the natural environment, the Se uptake ability of Se-rich rice cultivars was stronger than non-Se-responsive rice cultivar (GangYou 725).

### 3.2. Elemental Uptake and Recovery Rate

**Figure 2:**
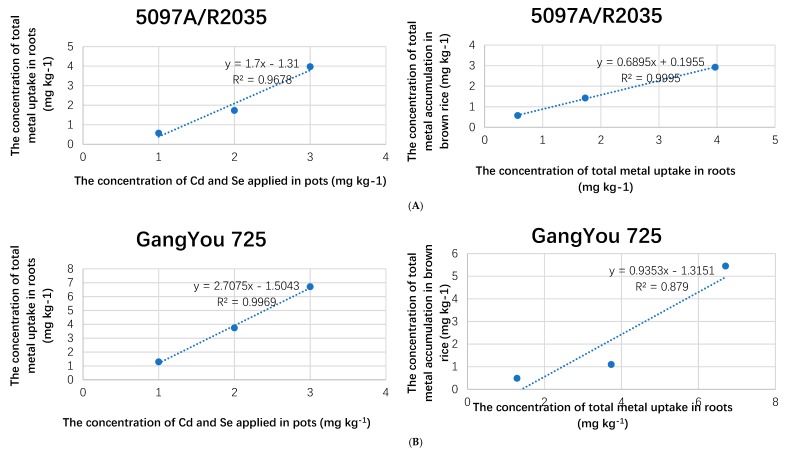

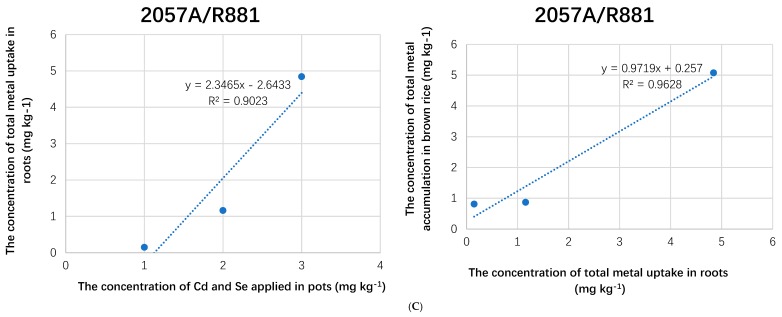
(**A**) The linear association between the different components of Se-enriched rice (5097A/R2035); (**B**) The linear association between the different components of non-Se-enriched rice (GangYou 725); (**C**) The linear association between the different components of Se-enriched rice (2057A/R881).

A glimpse of metal’s uptake (%) in rice tissues and varietal comparison can be seen in Supplementary Table S1. When different treatment levels were compared, an increasing trend in metal uptake was observed. Total metal concentrations of 14 mg and 30 mg were applied in pots at T_1_ and T_2_ treatment levels, while 5 mg of metal contents (Cd+Se) were found in T_0_ pot’s soil. The increased fertilizer treatment in soil bulk linearly increased the content of metal in rice tissues (Figure 2A–C). Peng et al. also found that the increased Cd contents in soil resulted in increased Cd concentration in plant shoots [27]. The metal uptake capability of GangYou725 roots was stronger (R^2^ = 0.99) (Figure 2A) with high metal contents of approximately 48% to 70% (Supplementary Table S1) than Se-rich rice cultivars, while the translocation of metal from roots to grain (R^2^ = 0.87) was not so effective in them (Figure 2B). 

Scientific review literature cited that only 70% of total heavy metal gains successful entry into the plant; it is likely that chelation, sequestration, cellular exclusion, and chemical modification restricts its intake [28,29]. The other possible reason could be the soil pH, which contributes as an important factor in the metal mobilization and uptake by roots. For instance, Zeng et al. (2011) documented significantly a negative relationship between pH and available heavy metals [30]. The high exogenous metal application modified the soil environment to acidic, especially in lower layers (Supplementary Table S2, T_2_). Low pH enhances metal extractability and bioavailability [31] and promotes its accumulation in rice tissues [32], especially at elevated stress level T_2_ (Supplementary Table S1, T_2_). Metals can enter root cells via certain channels, as well as transporters [33], which are manipulated by various sources viz., cultivar, nutrient levels and Cd concentration in soil [34]. About 5% to 20% of metal was found to be leached down into soil. Some proportion of metal could volatilize into the atmosphere, as disclosed by various reports [28,35]. Hence, in the present study, the metal proportion applied was not completely recovered in rice tissues and soil; it was probably volatilized into the surrounding atmosphere. The extent of volatilization varied among cultivars (Supplementary Table S1, T_1_~T_2_). Se can undergo subsequent changes into plastid, chloroplast, and root exudates, and can exit the plant in the less toxic form of dimethylselenide (DMSe) and dimethyldiselenide (DMDSe) into the surrounding environment [16,28]. In present investigation, sodium selenite was used as fertilizer, while a number of earlier studies have reported different findings, where the model plant favored the uptake of selenate over selenite because of its easy bioavailability in soil [36,37]. The varietal insights indicated that although non-Se rich rice recovered the maximum metal content from soil, it is informative to dissect the proportion of Cd and Se that became accumulated in rice tissues. 

### 3.3. Vegetative versus Reproductive Rice Tissues, Metal’s Uptake Trend

The actual metal content (mg kg^−1^) that accumulated in rice tissues at varying stress level can be seen in Supplementary Tables S3–S5. When compared rice vegetative parts (roots, stem, leaves and panicle; Supplementary Figures S1–S3), the roots and panicles were found to have maximum metal accumulation, followed by the stems and leaves (Table 3) [38]. The root exudates significantly restrict, sequestrate, and provide safe passages for Cd bioavailability and toxicity to plants, by manipulating the number and activity of rhizospheric microbes, redox potential, rhizosphere pH, and chelating capacity for ionic Cd [34]. Under natural conditions, the Se and Cd accumulation behaviour in Se-rich rice cultivars was panicle > roots > stem > leaves > soil and soil > panicle > roots > stem > leaves, respectively. The uptake of Se and Cd in non-Se-rich rice (GangYou 725) was in order; root > soil > panicle > leaves > stem, and panicle > roots > leaves > stem > soil, respectively (Supplementary Table S3). The exogenous fertilizer treatments (T_1_ and T_2_) altered the natural metal uptake response in GangYou 725: Se; root > panicle > stem > leaves > soil, and 5097A/R2035: Se; panicle > roots > stem > leaves > soil. The Cd uptake trend was nearly the same in rice cultivars as soil > panicle > roots > stem > leaves (Table 3). Se-rich rice cultivars utilized soil Se reserves and translocated Se linearly in edible parts of rice, while Cd sequestrated into the soil and roots (Supplementary Tables S4 and S5). The translocation of Cd ions within the plasmodesmata cells [39] enables their entry into the xylem cells, either in phytochelatins or organic acids form [40]. The passage might be responsible for the differential accumulation of metal in leaves, stems, panicles [41], and ultimately to the grain [42]. Uraguchi et al. observed the Cd accretion in shoots and grains of two rice cultivars (Habataki; high Cd content in grain and Sasanishiki; low Cd content in grain). Uraguchi attributed the high transpiration rate and high xylem loading ability of Habataki genotypes as the reason for high Cd accumulation [43]. The high Cd stress can affect the physiology and reduce dry weight of plants, with an elevated accretion of Cd content in grain fractions [44]. The high concentration of Cd content in GangYou725 is probably due to the soil application of metal treatment during critical growth phases (just prior to the booting stage) and slow Cd mobilization in soil, as the plant uptake ample amount of nutrients at developmental stages [45] might be the reason for the high Cd content in non-Se rich rice.

Then, the panicle was split into its components (panicle straw, husk, rice bran, endosperm, and embryo) to critically analyze the metal accumulation trend in each part. The trend in the accumulation behavior of Se and Cd in panicle components was: rice bran > husk > embryo > panicle straw > endosperm, and rice bran > husk > panicle straw > embryo > endosperm, respectively (Table 3). It was found that rice bran can store half of the total metal, while the remaining half of it gets stored into the husk, endosperm, and the embryo [46]. Rice bran accounts for approximately 1% to 2% of the total grain [47]. During dough and yellow ripe stage development, an aleurone layer forms on surface of the endosperm cells on the eighth to ninth day after the blooming of the rice flower, acting as an intermediate passage for absorption and translocation of nutrients left on the surface layer of endosperm cells, and playing significant role in the storage of proteins, starch, lipids, and mineral elements [48]. Since rice bran is more eminent, it can store more metal contents than polished rice because of the starchy nature of endosperm cells, while the translocation of metals might be restricted to husk and rice bran. At the beginning of the caryopsis after seven days, the accumulation of starch in endosperm cells increases gradually, and the cells eventually lose their activity probably between 14–18 days [48,49], resulting in plentiful metal content (75%) absorbed by the rice bran and husk (probably inorganic) that cannot be transferred to endosperm [50,51,52]. Panicle straw linked the grains with stem, and no obvious metal’s interactive study on this component in rice is available. Hence, after determining the metal content in this part, it was found that panicle straw was enriched with more Cd rather than Se when compared with stem, particularly in non-Se-rich rice (Table 3). During grain maturity stage, the remobilization of plant’s reserves to grain may result in the localization of high Cd concentration in panicle straw, which is probably due to high water-use efficiency [53]. The embryo and endosperm are the ultimate produce consumed for edible purposes. The purity and quality of the grain’s endosperm ad embryo are very important, as they are directly linked to human health. However, a study needs to be explored for the health-related risks and milling losses for different levels of metal treatment.

### 3.4. Health Risks and Milling Losses in Cd and Se-Treated Rice

The major concern for the utilization of rice is related to brown rice, particularly its embryo and endosperm [54]. These components need to be discussed critically in order to assess the nutritional aspects. The approximate assimilation of Cd is considered hazardous for human health if accumulated beyond 0.2 mg kg^−1^ in polished rice (GB 2762-2017) [55]. Under natural environment (T_0_) and low-stress treatment (T_1_), the Se content in embryo and endosperm of Se-rich rice cultivars were within the range of national standards for a rich Se-paddy, i.e., 0.04~0.3 mg kg^−1^ (GB/T 22499-2008). Meanwhile, Cd contents were approaching the traditional threshold level for statistical significance—that is, about 0.2 mg kg^−1^ (GB 2762-2017). The percent of Se milling loss due to polishing operation was heightened over 24-fold to 28-fold more than Cd (6~13%) in Se-rich rice cultivars, indicating that most of the metal resides in rice bran (Supplementary Tables S3 and S4). The percent of Cd milling loss in non-Se rich (GangYou 725) rice was 16%. 

Usually, the introduction of heavy metals in plants generated reactive oxygen species (ROS) and oxidative stress, but the level of metal greatly influences plant functional response and both enzyme and non-enzyme antioxidant systems to fight against Cd toxicity [56]. At low levels, some of the specific trace elements aid the plant in heavy metal-mediated stress mitigation, but at elevated levels, the same metal can have an inverse effect on functional response of plant [57]. In the present study, rice cultivars were found to be vulnerable to metal’s accumulation in embryo and endosperm; the metal content was over the permitted range, indicating a hyperaccumulation of Se and Cd at elevated stress treatment level T_2_ (Supplementary Table S5). The milling and polishing operation may not be beneficial at this level. This probably implied that high Cd and Se concentration in soil lead to the ample uptake of metals in rice tissues [54]. Therefore, the rice cultivation at 2 mg kg^−1^ Cd-affected soil may actually impose health risks by Cd accretion in polished rice. The 1 mg kg^−1^ Se application mitigates Cd toxicity, but the accumulation of Se in rice tissues was heightened over the permitted range in polished rice. This might be a serious threat for human health, as rice is the major food crop that could be a source for metal’s entrance into food chain.

### 3.5. Effect of Heat Units on Rice Nutritional Status

The impact of heat units on the nutritional contents of rice was observed. The three materials under investigation were sown at the same date, which tend to mature at different times. The amount of heat units consumed by the moderate Se-rich rice (2057A/R881) and non-Se rich rice (GangYou 725) were nearly the same while achieving a specific stage of heading, flowering, and 50% maturity till harvesting. The trend in the high Se-rich rice group (5097A/R2035) was different. The Se-rich rice (5097A/R2035) consumes a lower amount of heat units than the other rice cultivars (Table 4). Moreover, it was observed that the contents of Se and Cd were under the hazardous levels, even at elevated Cd stress levels in high Se-rich rice (5097A/R2035) (Supplementary Tables S4 and S5). Meanwhile, moderate and low Se responsive rice cultivars tend to mature late, accumulating more metal contents, and causing Se and Cd hypertoxic effects. 

The impact of warming on the uptake of Cd was observed by Ge at different sowing times [58,59]. It was observed that late sowing increases the uptake of Cd in plants [59,60]. However, in the present study, material that was sown at the same time tends to mature at different times. The materials that matured late accumulated more metal content (Supplementary Table S5). Hence, the proper time of planting, harvesting, and adequate management practices are recommended to avoid health-associated risks [26,61]. The current Se contingence is mainly due to the different sensitivity of varietal responses to Se. The process of Se assimilation and metabolism by the roots runs antagonistically to that of the leaves, as there is enough time for Se assimilation and its transformation into organic form within the growth period of the crop [62]. The comprehensive analysis designs and contains detailed discussion about the accumulation and trending behaviour of Cd and Se, which open new insights and provide a better understanding of the functional response of rice components.

## 4. Conclusions 

The current study disclosed that Se has significant effects on Cd uptake and mitigation.

- The increased fertilizer treatment in soil bulk linearly increased the metal contents in rice grain.
- Rice tissues uptake 50–70% of metal applied, while 5–20% leached down into the soil.
- The Cd concentration in polished rice of Se-rich rice was below the threshold level of Cd (0.2 mg kg^−1^), when Se was applied at a rate of 0.4 mg kg^−1^ at tillering stage.
- Panicles and rice bran accumulated a maximum of metal contents. At low Cd stress (1 mg kg^−1^), the metal accumulation in rice tissues was within a safe range, while at high Cd stress (2 mg kg^−1^), a hyper accumulative Se effect was observed in polished rice.
- The late matured rice cultivars consumed more heat units, and more metal contents were assessed in them.

The field study on rice nutritional aspects scrutinizes the associated health risks and utilization of rice for Cd-affected soil. However, further molecular studies are required in order to completely access the inverted Se accumulation behavior in rice tissues at high Cd soil stress.

## Figures and Tables

**Table 1:** The basic physicochemical properties of test soil.

Index	Contents (mg kg^−1^ Dry Soil)
pH	5.98
OM	32,870
Total N	172
Total P	1960
Total Se	0.3242
Total Cd	0.0912

OM = Organic Matter, N = Nitrogen, P = Phosphorus, Se = Selenium, Cd = Cadmium.

**Table 2:** Stress treatment groups used in the study.

Treatment	Na_2_SeO_3_ (mg kg^−1^ Dry Soil)	CdCl_2_.2½H_2_O (mg kg^−^^1^ Dry Soil)	Remarks
T0 (Control)	-	-	Adequate all
T1	0.4	1	Medium Stress
T2	1	2	High Stress

Note: T_0_ (Natural soil conditions), T_1_ (Se; 0.4 mg kg**^−^**^1^, Cd; 1 mg kg**^−^**^1^), T_2_ (Se; 1 mg kg**^−^**^1^, Cd; 2 mg kg**^−^**^1^).

**Table 3:** Two-factor factorial analysis design (varieties × stress, treatment × stress interaction effects all pairwise possible combinations).

Interaction Effects		Soil	Roots	Stem	*Leaves*	*Panicle*	*Panicle Straw*	*Husk*	*Rice Bran*	*Embryo*	*Endosperm*
**Varieties vs. Stress**											
	**V1 × Se**	0.220 ^b^	2.090 ^b^	1.039 ^a^	0.689 ^a^	3.399 ^a^	0.791 ^a^	0.971 ^a^	1.046 ^a^	0.458 ^abc^	0.132 ^a^
	**V2 × Se**	0.914 ^ab^	3.236 ^a^	1.018 ^a^	0.975 ^a^	2.968 ^a^	0.525 ^ab^	0.658 ^ab^	0.820 ^a^	0.608 ^ab^	0.355 ^a^
	**V3 × Se**	0.738 ^ab^	2.969 ^ab^	1.033 ^a^	0.962 ^a^	3.068 ^a^	0.505 ^ab^	0.669 ^ab^	0.855 ^a^	0.666 ^a^	0.370 ^a^
	**V1 × Cd**	2.428 ^a^	0.277 ^c^	0.190 ^b^	0.155 ^a^	0.369 ^c^	0.092 ^b^	0.085 ^c^	0.106 ^b^	0.044 ^c^	0.041 ^a^
	**V2 × Cd**	1.887 ^ab^	0.674 ^c^	0.152 ^b^	0.199 ^a^	1.080 ^b^	0.225 ^b^	0.296 ^bc^	0.281 ^b^	0.146 ^bc^	0.131 ^a^
	**V3 × Cd**	1.969 ^a^	0.561 ^c^	0.133 ^b^	0.152 ^a^	1.004 ^bc^	0.181 ^b^	0.266 ^bc^	0.288 ^b^	0.153 ^bc^	0.115 ^a^
**Treatments vs. Stress**											
	**T0 × Se**	0.288 ^b^	0.517 ^c^	0.218 ^b^	0.167 ^b^	0.695 ^bc^	0.127 ^b^	0.135 ^b^	0.254 ^bc^	0.135 ^b^	0.043 ^b^
	**T0 × Cd**	0.230 ^b^	0.277 ^c^	0.121 ^b^	0.110 ^b^	0.480 ^c^	0.126 ^b^	0.128 ^b^	0.101 ^c^	0.066 ^b^	0.058 ^b^
	**T1 × Se**	0.474 ^b^	2.433 ^b^	0.284 ^b^	0.212 ^b^	1.197 ^b^	0.190 ^b^	0.262 ^b^	0.576 ^b^	0.124 ^b^	0.045 ^b^
	**T1 × Cd**	0.935 ^b^	0.674 ^c^	0.240 ^b^	0.213 ^b^	1.040 ^bc^	0.204 ^b^	0.289 ^b^	0.393 ^bc^	0.079 ^b^	0.074 ^b^
	**T2 × Se**	1.110 ^b^	5.334 ^a^	2.588 ^a^	2.246 ^a^	7.543 ^a^	1.505 ^a^	1.902 ^a^	1.892 ^a^	1.473 ^a^	0.769 ^a^
	**T2 × Cd**	5.123 ^a^	0.561 ^c^	0.113 ^b^	0.184 ^b^	0.933 ^bc^	0.168 ^b^	0.231 ^b^	0.181 ^bc^	0.197 ^b^	0.155 ^b^
**S. E**		0.6270	0.3379	0.2791	0.3777	0.2391	0.2015	0.1917	0.1688	0.1737	0.1548
**LSD (0.05)**		1.7408	0.9383	0.7748	1.0487	0.6638	0.5595	0.5321	0.4685	0.4822	0.4299
**Interaction Effects**											
(Varieties × Stress)		*	*	*	ns	*	*	*	*	*	ns
(Treatment × Stress)		*	*	*	*	*	*	*	*	*	*

Note: V1: 5097A/R2035, V2: GangYou 725, V3: 2057A/R881. T_0_; 0, T_1_ (0.4 + 1) and T_2_ (1 + 2) refer to mg Se + Cd kg^−1^ respectively. Means do not share the same letters in the column differ significantly at *p* ≤ 0.05; * = Significant, ns = non-significant, S.E; Standard Error, LSD; least significant differences (5% probability).

**Table 4:** Number of days and amount of heat units consumed by rice to achieve respective stage.

	**Estimated Date of the Respective Stage**								
**Varieties**	**Sowing**	**Transplanting**	**Stress Treatment**	**Tillering**	**50% Heading**	**Flowering**	**50% Maturity**	**Maturity**	**Harvesting**
5097A/R2035	13/04	23/05	03/06	22/07	14/08	21/08	27/08	04/09	12/09
GangYou 725	13/04	23/05	04/06	18/07	08/08	14/08	21/08	31/08	06/09
2057A/R881	13/04	23/05	04/06	28/07	16/08	22/08	28/08	05/09	11/09
	**Days took to achieve the respective stage**								
		**Transplanting**	**Stress Treatment**	**Tillering**	**50% Heading**	**Flowering**	**50% Maturity**	**Maturity**	**Harvesting**
5097A/R2035	--	40	51	95	116	122	129	139	145
GangYou 725	--	40	50	99	122	129	135	143	151
2057A/R881	--	40	51	105	124	130	136	144	150
	**Heat units consumed by rice to achieve the respective stage**								
		**Transplanting**	**Stress Treatment**	**Tillering**	**50% Heading**	**Flowering**	**50% Maturity**	**Maturity**	**Harvesting**
5097A/R2035	--	408	563	1224	1598.5	1702.5	1818.5	1981.5	2054.5
GangYou 725	--	408	550.5	1292.5	1702.5	1818.5	1916.5	2031	2142
2057A/R881	--	408	563	1398	1736	1836	1938	2042	2126.5

Note: High Se-rich rice: 5097A/R2035, Non-Se rich rice: GangYou 725, Moderate Se-rich rice: 2057A/R881. The heat units were assessed by estimating the daily differences in temperature and then compiling them to know the exact amount of heat units consumed by rice while reaching each stage (refer to Section 2.7 for method).

## Supplementary Materials

The following are available online at https://www.mdpi.com/2218-273X/9/6/247/s1.
