# Supplementary material for: Cross-Talk between Cadmium and Selenium at Elevated Cadmium Stress Determines the Fate of Selenium Uptake in Rice
Source: Biomolecules. 2019 Jun 24;9(6):247. doi: 10.3390/biom9060247 (PMC6627080; doi:10.3390/biom9060247)
Supplement: Supplementary file 1 [file biomolecules-09-00247-s001.pdf]

Supplementary Table A The metal uptake and recovery rate in rice.

Treatment	Total metal applied in soil (10kg)	Total metal uptake (%)			Metal recovered in rice tissues (%)			Metal leached down in soil (%)		
		V1	V2	V3	V1	V2	V3	V1	V2	V3
T_0	5 mg kg^{-1}	42.79	61	26.86	38.21	48.21	23	4.57	12.79	3.86
T_1	14 mg kg^{-1}	69.40	73.80	43.00	62.80	58.80	33.60	6.60	15.00	9.40
T_2	30 mg kg^{-1}	77.07	90.27	81.53	53.83	70.73	67.43	23.23	19.53	14.10

Note: V1: 5097A/R2035, V2: GangYou 725, V3: 2057A/R881, Metal contents (Cd+Se)

Supplementary Table B. Soil pH under different treatment levels in pots.

Treatment	Soil Depth	V1	V2	V3
T_0	10 cm	5.96	5.78	5.80
	20 cm	6.21	6.02	6.12
T_1	10 cm	5.36	5.44	5.75
	20 cm	6.41	6.13	6.19
T_2	10 cm	5.43	5.35	5.52
	20 cm	5.91	5.86	5.65

Note: V1: 5097A/R2035, V2: GangYou 725, V3: 2057A/R881

Supplementary Table C. Accumulation behaviour in different rice tissues under natural condition (T_0).

**Cd: 0.091 mg/kg**	Se-rich rice (5097A/R2035)		Non-Se rich rice 725		Se-rich 2057A/881	
**Se: 0.32 mg/kg**	Se	Cd	Se	Cd	Se	Cd
Soil (10 cm)	0.039	0.123	0.382	0.065	0.048	0.214
Soil (20 cm)	0.072	0.098	0.259	0.047	0.064	0.143
Total Soil	0.111	0.221	0.641	0.112	0.112	0.357
Root	0.569	0.175	0.892	0.398	0.091	0.058
Stem	0.745	0.112	0.030	0.155	0.077	0.098
Leave	0.408	0.099	0.066	0.186	0.027	0.045
Panicle	0.775	0.255	0.506	0.707	0.805	0.480
Panicle Straw	0.077	0.088	0.182	0.213	0.123	0.079
Husk	0.131	0.053	0.121	0.211	0.154	0.121
Bran	0.308	0.044	0.175	0.118	0.280	0.141
Embryo	0.191	0.040	0.019	0.069	0.195	0.090
Endosperm	0.069	0.029	0.009	0.096	0.053	0.049
Total plant	2.498	0.641	1.495	1.447	0.999	0.681

Note: T_0 Control (Natural soil conditions), T_1 (Se; 0.4 mgkg-1, Cd; 1 mgkg-1), T_2 (Se; 1 mgkg-1, Cd; 2 mgkg-1).

Supplementary Table D. Accumulation behaviour in different rice tissues under stress treatment (T_1)

**Cd: 1 mg/kg**	Se-rich rice (5097A/R2035)		Non-Se rich rice 725		Se-rich 2057A/881	
**Se: 0.4 mg/kg**	Se	Cd	Se	Cd	Se	Cd
Soil (10 cm)	0.054	0.369	0.382	0.841	0.048	0.171
Soil (20 cm)	0.086	0.131	0.259	0.312	0.064	0.253
Total Soil	0.14	0.5	0.641	1.153	0.112	0.424
Root	1.732	0.275	2.784	0.953	1.091	0.067
Stem	0.302	0.235	0.177	0.243	0.132	0.084
Leave	0.389	0.116	0.124	0.262	0.256	0.127
Panicle	1.82	0.48	0.886	1.321	0.899	0.565
Panicle Straw	0.17	0.118	0.2	0.247	0.15	0.09
Husk	0.23	0.093	0.278	0.387	0.196	0.163
Bran	0.98	0.155	0.374	0.513	0.28	0.09
Embryo	0.33	0.077	0.021	0.081	0.195	0.141
Endosperm	0.11	0.037	0.013	0.093	0.078	0.081
Total plant	4.243	1.106	3.971	2.779	2.378	0.843

Note: T_0 Control (Natural soil conditions), T_1 (Se; 0.4 mgkg-1, Cd; 1 mgkg-1), T_2 (Se; 1 mgkg-1, Cd; 2 mgkg-1).

Supplementary Table E. Accumulation behaviour in different rice tissues under stress treatment (T_2).

**Cd: 2 mg/kg**	Se-rich rice (5097A/R2035)		Non-Se rich rice 725		Se-rich 2057A/881	
**Se: 1 mg/kg**	Se	Cd	Se	Cd	Se	Cd
Soil (10 cm)	0.197	6.330	1.159	4.193	0.070	4.021
Soil (20 cm)	0.212	0.235	0.301	0.205	0.065	0.071
Total Soil	0.409	6.565	1.461	4.398	0.135	4.091
Root	3.969	0.383	6.033	0.672	4.478	0.364
Stem	2.071	0.223	2.847	0.059	3.439	0.104
Leave	1.27	0.252	2.735	0.151	2.285	0.249
Panicle	7.603	0.373	7.513	1.213	8.719	0.598
Panicle Straw	2.128	0.070	1.194	0.217	2.219	0.133
Husk	2.553	0.109	1.577	0.292	1.770	0.121
Bran	1.850	0.119	1.913	0.212	2.731	0.133
Embryo	0.853	0.016	1.784	0.288	1.425	0.120
Endosperm	0.219	0.059	1.045	0.204	0.574	0.091
Total plant	14.913	1.231	19.128	2.095	18.921	1.315

Note: T_0 Control (Natural soil conditions), T_1 (Se; 0.4 mgkg-1, Cd; 1 mgkg-1), T_2 (Se; 1 mgkg-1, Cd; 2 mgkg-1).

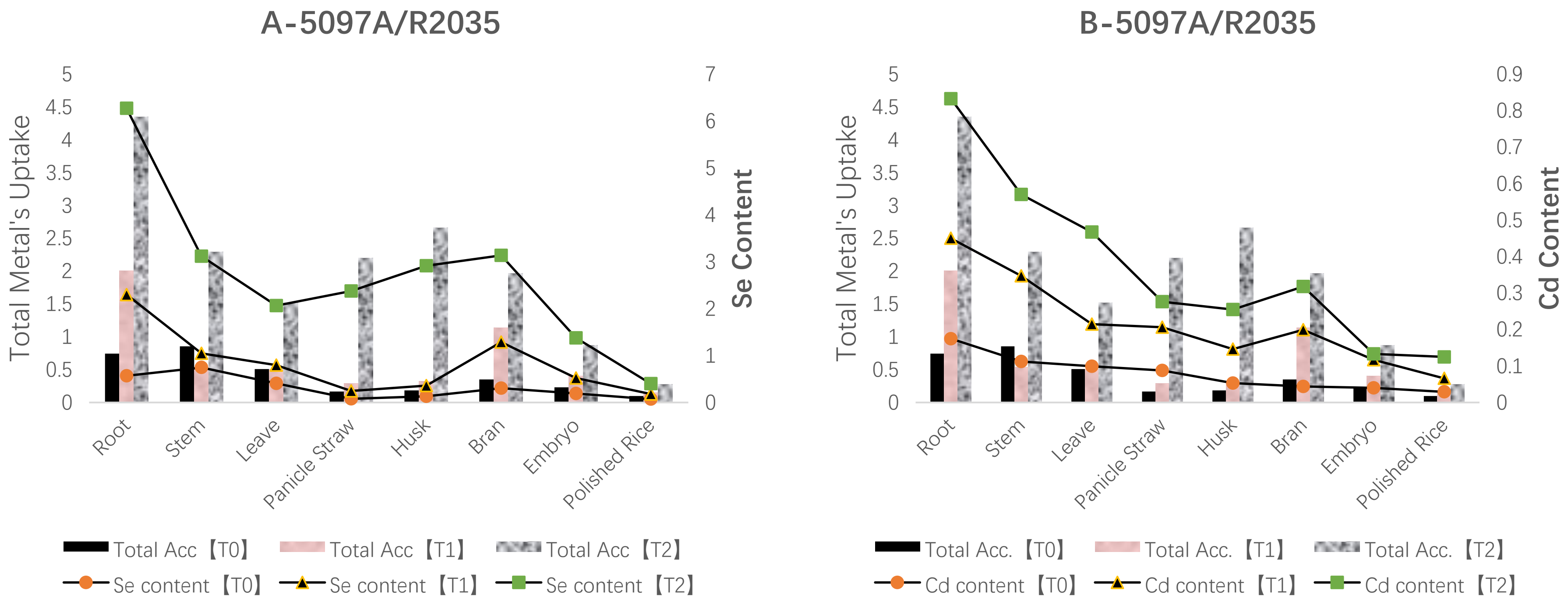

Supplementary Figure 1. The comparison of Se and Cd contents uptake by rice total produce under different treatment levels (T_0, T_1, T_2) in Se-enriched rice 5097A/R2035. Note: T_0 (Natural soil conditions), T_1 (Se; 0.4 $mgkg^{-1}$, Cd; 1 $mgkg^{-1}$), T_2 (Se; 1 $mgkg^{-1}$, Cd; 2 $mgkg^{-1}$), Total Acc: Total metal's (Cd+Se) accumulation $mgkg^{-1}$. Legends; X-axis; rice different components comparison, Y-axis (Left); The total metal's (Se+Cd) uptake at T_0, T_1 and T_2 (indicated with histogram), Y-axis (Right side); The actual contents of Cd and Se got accumulated in different parts at T_0, T_1, T_2 (indicated with trending line).

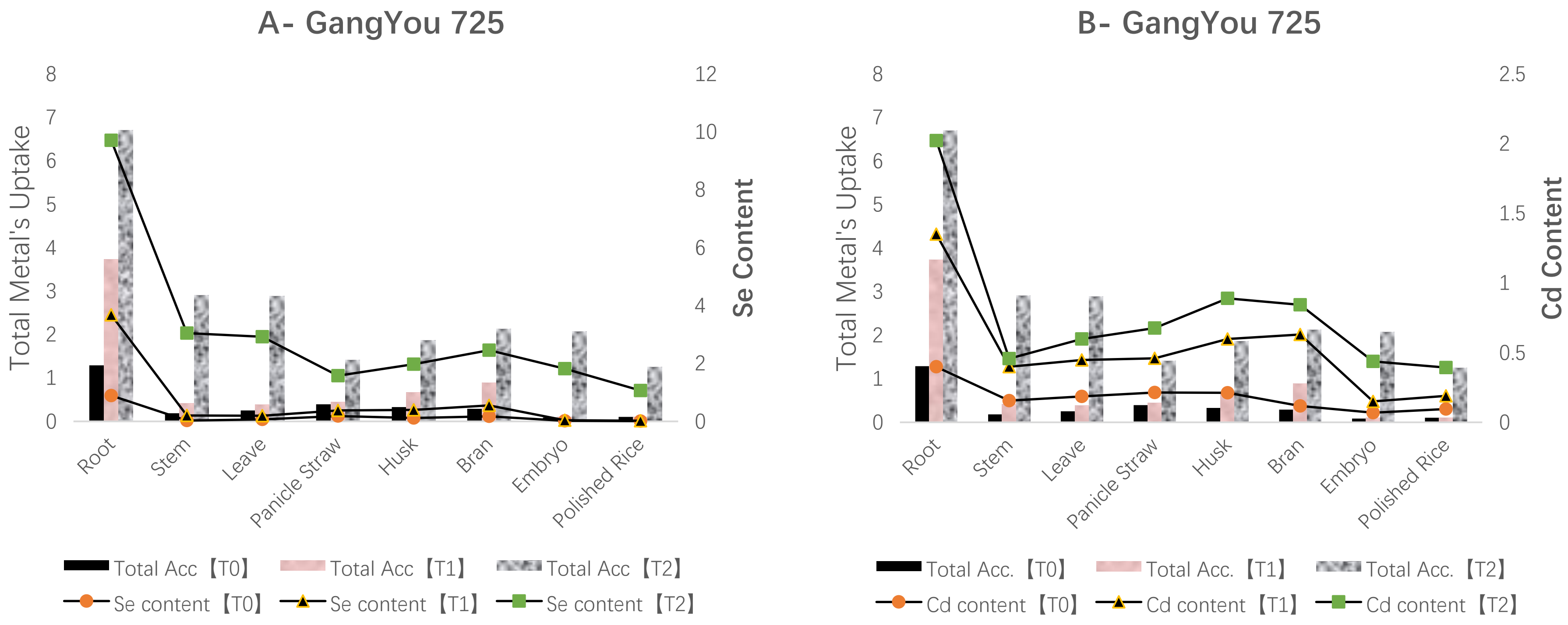

Supplementary Figure 2. The comparison of Se and Cd contents uptake by rice total produce under different treatment levels (T_0, T_1, T_2) in non-Se-enriched rice GangYou 725.

Note: T_0 (Natural soil conditions), T_1 (Se; 0.4 mgkg^{-1}, Cd; 1 mgkg^{-1}), T_2 (Se; 1 mgkg^{-1}, Cd; 2 mgkg^{-1}), Total Acc: Total metal's (Cd+Se) accumulation mgkg^{-1}. Legends; X-axis; rice different components comparison, Y-axis (Left); The total metal's (Se+Cd) uptake at T_0, T_1 and T_2 (indicated with histogram), Y-axis (Right side); The actual contents of Cd and Se got accumulated in different parts at T_0, T_1, T_2 (indicated with trending line).

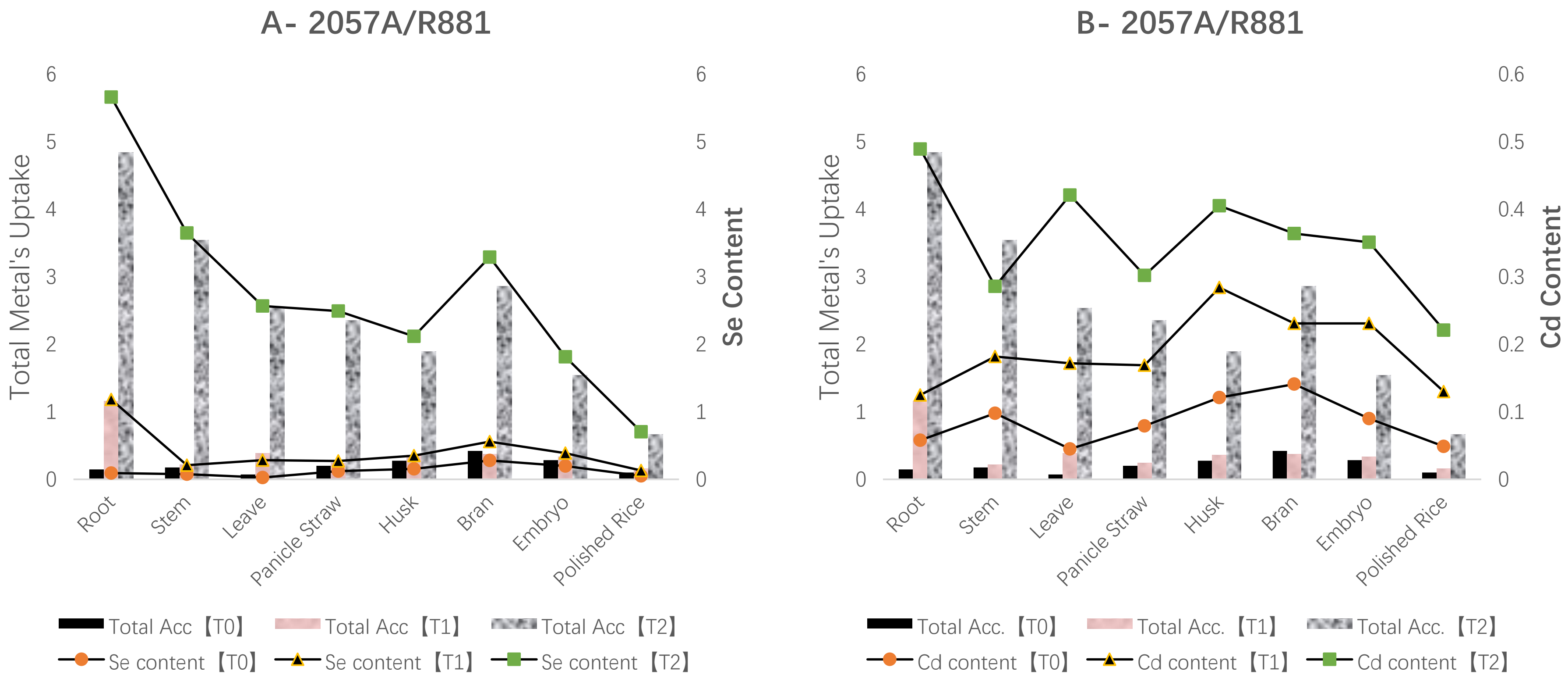

Supplementary Figure 3. The comparison of Se and Cd contents uptake by rice total produce under different treatment levels (T_0, T_1, T_2) in Se-enriched rice 2057A/R881. Note: T_0 (Natural soil conditions), T_1 (Se; 0.4 $mgkg^{-1}$, Cd; 1 $mgkg^{-1}$), T_2 (Se; 1 $mgkg^{-1}$, Cd; 2 $mgkg^{-1}$), Total Acc: Total metal's (Cd+Se) accumulation $mgkg^{-1}$. Legends; X-axis; rice different components comparison, Y-axis (Left); The total metal's (Se+Cd) uptake at T_0, T_1 and T_2 (indicated with histogram), Y-axis (Right side); The actual contents of Cd and Se got accumulated in different parts at T_0, T_1, T_2 (indicated with trending line).
